# Psychotropic drug-induced genetic-epigenetic modulation of *CRTC1* gene is associated with early weight gain in a prospective study of psychiatric patients

**DOI:** 10.1186/s13148-019-0792-0

**Published:** 2019-12-26

**Authors:** Aurélie Delacrétaz, Anaïs Glatard, Céline Dubath, Mehdi Gholam-Rezaee, Jose Vicente Sanchez-Mut, Johannes Gräff, Armin von Gunten, Philippe Conus, Chin B. Eap

**Affiliations:** 1 Unit of Pharmacogenetics and Clinical Psychopharmacology, Centre for Psychiatric Neuroscience, Department of Psychiatry, Lausanne University Hospital, University of Lausanne, Prilly, Switzerland; 2 Centre of Psychiatric Epidemiology and Psychopathology, Department of Psychiatry, Lausanne University Hospital, University of Lausanne, Prilly, Switzerland; 3 Laboratory of Neuroepigenetics, Brain Mind Institute, School of Life Sciences, École Polytechnique Fédérale de Lausanne, Lausanne, Switzerland; 4 Service of Old Age Psychiatry, Department of Psychiatry, Lausanne University Hospital, University of Lausanne, Prilly, Switzerland; 5 Service of General Psychiatry, Department of Psychiatry, Lausanne University Hospital, University of Lausanne, Prilly, Switzerland; 6 Institute of Pharmaceutical Sciences of Western Switzerland, University of Geneva, Geneva, Switzerland

**Keywords:** Early weight gain, Psychotropic drugs, CRTC1, Methylation, Psychiatric population

## Abstract

**Background:**

Metabolic side effects induced by psychotropic drugs represent a major health issue in psychiatry. CREB-regulated transcription coactivator 1 (*CRTC1*) gene plays a major role in the regulation of energy homeostasis and epigenetic mechanisms may explain its association with obesity features previously described in psychiatric patients. This prospective study included 78 patients receiving psychotropic drugs that induce metabolic disturbances, with weight and other metabolic parameters monitored regularly. Methylation levels in 76 *CRTC1* probes were assessed before and after 1 month of psychotropic treatment in blood samples.

**Results:**

Significant methylation changes were observed in three *CRTC1* CpG sites (i.e., cg07015183, cg12034943, and cg 17006757) in patients with early and important weight gain (i.e., equal or higher than 5% after 1 month; FDR *p* value = 0.02). Multivariable models showed that methylation decrease in cg12034943 was more important in patients with early weight gain (≥ 5%) than in those who did not gain weight (*p* = 0.01). Further analyses combining genetic and methylation data showed that cg12034943 was significantly associated with early weight gain in patients carrying the G allele of rs4808844A>G (*p* = 0.03), a SNP associated with this methylation site (*p* = 0.03).

**Conclusions:**

These findings give new insights on psychotropic-induced weight gain and underline the need of future larger prospective epigenetic studies to better understand the complex pathways involved in psychotropic-induced metabolic side effects.

## Background

Metabolic diseases such as obesity or dyslipidemia arise from a complex interplay between genetic and environmental factors. During the last two decades, many efforts were put into understanding how genetic and environmental factors interact in the development of cardiometabolic diseases. Recently, epigenetic mechanisms have been proposed to link the genetic background with environmental influences, placing important expectations on the potential to unravel mechanisms of cardiometabolic diseases [1–6]. In psychiatry, patients suffering from schizophrenia, bipolar disorder, and major depressive disorders have a reduced life expectancy of 10–15 years compared to individuals from the general population [7, 8], which is mainly attributable to cardiovascular diseases resulting from the metabolic syndrome [9]. Multiple risk factors implying complex mechanisms may explain this excessive susceptibility for developing metabolic diseases, including psychiatric disease-related factors, unhealthy lifestyle, genetic susceptibilities, and adverse effects of treatment [10–12]. Thus, the use of psychotropic medications such as antipsychotics (most atypical but also some typical), mood stabilizers (e.g., lithium), and some antidepressants (e.g., mirtazapine) can increase the risk of metabolic disorders, including obesity and dyslipidemia [13, 14]. Metabolic side effects induced by psychotropic drugs result from an appetite increase driven by multiple mechanisms, including pharmacodynamic affinities as well as modifications in the transcription of some hormones involved in energy homeostasis [15, 16]. Although epigenetic mechanisms underlying side effects induced by psychotropic treatments are yet very poorly understood, some studies have determined methylation changes associated with atypical antipsychotics [17–19] and mood stabilizers [20–22], some of which were linked with the recovery of molecular aberrations observed in patients with psychiatric diseases [23]. Concerning metabolic side effects, two recent studies have drawn attention to associations between certain methylation sites and insulin resistance [24, 25]. However, it is unknown whether methylation modulations are tissue-specific and/or reversible. More importantly, epigenetic studies conducted in longitudinal settings are lacking.

Worsening of the metabolic condition may develop early during treatment with psychotropic drugs inducing metabolic disturbances [26–29] and may initiate a steady process leading to cardiometabolic diseases in the long term. This underlines the importance to prospectively monitor metabolic parameters in particular during the first months of treatment [30]. Of note, some studies using prospective weight data determined that a weight gain of 5% or more during the first month of treatment could robustly predict subsequent important weight gain [26] and metabolic syndrome [31].

Over the last decade, pharmacogenetics of psychotropic-induced weight gain has been extensively studied using candidate gene approaches, in particular within dopamine and serotonin receptors [32, 33]. In addition, numerous single nucleotide polymorphisms (SNPs) within genes involved in other pathways of metabolism (e.g., in enzymes, receptors, or transcriptional coactivators involved in food intake homeostasis) were also associated with weight gain in psychiatric patients receiving psychotropic drugs [34–38]. In particular, animal and human studies have identified the CREB-regulated transcription coactivator 1 (*CRTC1*) as an interesting candidate gene. Thus, mice lacking this gene developed obesity and other metabolic complications under normal diets [39–41]. Furthermore, while becoming obese, *CRTC1* knockout male mice on a normal chow diet were hyperphagic and had alterations in the expression of orexigenic and anorexigenic genes, underlining the key role of *CRTC1* in the regulation of food intake [42]. Interestingly, other studies showed that *CRTC1* knockout mice exhibit neurobehavioral endophenotypes related to mood disorders, depression-related behavior, and a blunted behavioral response to antidepressants [43]. Thus, growing evidence supports the hypothesis that obesity and depression may originate from shared biological pathways [44–46]. In human, we showed that a missense mutation in *CRTC1* (i.e., rs3746266A>G) is associated with body mass index in psychiatric patients treated with weight gain-inducing psychotropic drugs and in the general population [38]. Later on, a population-based study demonstrated that the influence of this SNP on obesity features was exclusively applicable in individuals with lifetime depression [47]. Finally, in a recent genome-wide association meta-analysis on body fat percentage in more than 100,000 individuals from the general population, *CRTC1* reached genome-wide significance and epigenetic mechanisms were suggested to explain this association [48].

These abovementioned aspects prompted us to investigate the influence of psychotropic drugs on *CRTC1* epigenetic modulation and to determine whether such modulations are associated with the risk of gaining weight and/or with *CRTC1* genetic polymorphism. For this purpose, methylation levels in 76 *CRTC1* probes were measured in DNA extracted from blood samples drawn in psychiatric patients before the initiation of weight gain inducing psychotropic drugs and after the first month of treatment, using each patient as his own control to dissociate the epigenetic effect from the genetic background. Data were compared between two groups of patients with extreme phenotypes, i.e., those developing early (after 1 month) and important (≥ 5%) weight gain (considered as “cases”) and those with no or minimal weight change (considered as “controls”).

## Results

### Demographics and CRTC1 methylation sites

The course of methylation levels during the first month of psychotropic treatment in 78 patients was investigated in 76 *CRTC1* probes (as listed in Additional file 1: Table S1). Median age was 37 years (IQR = 27–51 years), half of the patients were men (*n* = 39, 50%) and half of the patients smoked (*n* = 39, 50%) (Table 1). Psychotic disorders were the most frequent diagnosis (*n* = 35, 45%) and quetiapine was the most frequently prescribed psychotropic drug (*n* = 23, 30%). Half of the patients received psychotropic drugs with a moderate propensity for inducing weight gain (i.e., lithium, mirtazapine, quetiapine or risperidone, *n* = 42, 54%), while one-third received psychotropic drugs having a high risk for inducing weight gain (i.e., clozapine, olanzapine, or valproate, *n* = 24, 30%). One-third of patients was already overweight at baseline (*n* = 24, 31%) and this prevalence significantly increased during the first month of psychotropic treatment (*n* = 28, 36%, *p* = 0.04). A higher proportion of smokers (64% versus 37%; *p* = 0.02) was observed in control patients (i.e., patients without weight gain during the first month of treatment) than in case patients (i.e., patients with important weight gain during the same period). As expected, the proportion of overweight significantly increased in case patients (from 26% at baseline to 36% during the first month (*p* = 0.04), whereas it remained stable in control patients (37% in both treatment periods)). Of note, reported appetite (ranging from weak, moderate, medium, elevated and very elevated and collected at baseline and after the first month of psychotropic treatment) was significantly increased during the first month of treatment (*n* = 52; *p* = 0.048; data not shown).

**Table 1:** Demographic and clinical parameters of patients without and with early weight gain

	*N*	All patients (*n* = 78)	Controls^1^ (*n* = 39)	Cases^1^ (*n* = 39)	*p* value^2^
Age, median (IQR), years	78	37 (27–51)	40 (28–56)	37 (25–50)	0.47
Men, *n* (%)	78	39 (50.0)	19 (48.7)	20 (51.3)	0.82
Smoking at baseline, *n* (%)	74	37 (50.0)	23 (63.9)	14 (36.8)	*0.02*
Diagnosis, *n* (%)	78				
Psychotic disorders		35 (44.9)	17 (43.6)	18 (46.2)	0.82
Schizoaffective disorders		9 (11.5)	5 (12.8)	4 (10.3)	0.72
Bipolar disorders		18 (23.1)	12 (30.8)	6 (15.4)	0.11
Depressive disorders		6 (7.7)	2 (5.1)	4 (10.3)	0.4
Organic disorders		3 (3.9)	1 (2.6)	2 (5.1)	0.56
Other		6 (7.7)	2 (5.1)	4 (10.3)	0.4
Not available		1 (1.3)	0	1 (2.6)	0.31
Psychiatric illness duration, median (IQR), years	65	6 (2–11)	8 (2–13)	6 (2–10)	0.42
Medication, *n* (%)	78				
Amisulpride		3 (3.9)	0	3 (7.7)	0.08
Aripiprazole		9 (11.5)	5 (12.8)	4 (10.3)	0.72
Clozapine		4 (5.1)	2 (5.1)	2 (5.1)	1
Lithium		8 (10.3)	5 (12.8)	3 (7.7)	0.46
Mirtazapine		3 (3.9)	2 (5.1)	1 (2.6)	0.56
Olanzapine		19 (24.4)	8 (20.5)	11 (28.2)	0.43
Quetiapine		23 (29.5)	13 (33.3)	10 (25.6)	0.46
Risperidone		8 (10.3)	3 (7.7)	5 (12.8)	0.46
Valproate		1 (1.3)	1 (2.6)	0	0.31
Medication groups, *n* (%)^3^	78				
Low propensity for WG		12 (15.4)	5 (12.8)	7 (18.0)	0.53
Moderate propensity for WG		42 (53.9)	23 (59.0)	19 (48.7)	0.36
High propensity for WG		24 (30.8)	11 (28.2)	13 (33.3)	0.62
Baseline BMI, median (IQR), kg/m^2^	77	22.3 (19.8–25.6)	23.5 (21.3–26.4)	21.9 (18.6–25.4)	0.1
BMI at first month, median (IQR), kg/m^2^	77	23.8 (21.2–26.7)	23.7 (21.6–26.7)	23.9 (20.6–27.0)	0.94
Overweight prevalence (BMI ≥ 25 < 30 kg/m^2^), n(%)	77				
Baseline		24 (31.2)	14 (36.8)	10 (25.6)	0.29
First month		28 (36.4)	14 (36.8)	14 (35.9)	0.93
*p* value^4^		*0.04*	1	*0.04*	
Obesity prevalence (BMI ≥ 30 kg/m^2^), *n* (%)	77				
Baseline		7 (9.1)	4 (10.5)	3 (7.7)	0.67
First month		9 (11.7)	4 (10.5)	5 (12.8)	0.75
*p* value^4^		0.16	1	0.16	
WG, median (IQR), %	78	3.8 (0–7.7)	0 (0–1.4)	7.7 (5.8–10.9)	*< 0.0001*

*BMI* body mass index, *WG* weight gain

^1^Patients whose weight gain during the first month of treatment was between 0 and 2.5% were considered as controls, whereas patients whose weight gain during the first month of treatment was equal or higher than 5% were considered as cases

^2^*p* values were calculated using Wilcoxon rank-sum tests for continuous variables and *χ*^2^ tests for categorical variables

^3^Amisulpride and aripiprazole were considered as drugs with a low propensity for WG; lithium, mirtazapine, quetiapine, and risperidone were classified in the group with moderate propensity for WG, whereas clozapine, olanzapine, and valproate were considered as having a high propensity for WG

^4^McNemar tests were conducted to test for the difference between overweight and obesity between baseline and the first month of treatment.

Italic indicates significant *p* values

Pairwise *t* tests showed that during the first month of treatment, methylation levels in five probes localized in the *CRTC1* body region were significantly modified (Table 2). Thus, cg21310814, cg07015183, cg02961385, cg17006757, and cg22536770 significantly increased by 2, 0.8, 0.5, 0.9, and 0.4% (*n* = 78; FDR *p* value = 0.004, 0.004, 0.02, 0.048, and 0.048), respectively. When stratifying the population into early weight gain patient groups, no significant modification of *CRTC1* methylation was observed in patients whose weight remained stable, whereas methylation modulations in three sites were observed in patients with important early weight gain (i.e., 0.8, − 0.2, and 0.9% in cg07015183, cg12034943, and cg17006757; *n* = 39; FDR *p* value = 0.02, 0.02, and 0.02, respectively). Additional file 1: Figure S1 illustrates methylation levels of the abovementioned *CRTC1* probes according to the period of psychotropic treatment in the whole sample and in patients with early weight gain.

**Table 2:** Evolution of *CRTC1* methylation sites during the first month of psychotropic treatment

	Probe ID	β at baseline, median (IQR)	β after 1 month, median (IQR)	*p* value	FDR *p* value^1^
All patients (*n* = 78)	cg21310814	81.7 (78.5–84.3)	83.7 (81.2–84.9)	0.00005	0.004
	cg07015183	88.3 (87–89.5)	89.1 (88–90.3)	0.0001	0.004
	cg02961385	94.7 (94.2–95.4)	95.2 (94.7–96)	0.0009	0.02
	cg17006757	82.8 (80.4–84.2)	83.7 (82–85.3)	0.003	0.048
	cg22536770	94.1 (93.3–94.7)	94.5 (93.9–95.1)	0.003	0.048
Controls (*n* = 39)	None				
Cases (*n* = 39)	cg07015183	88.3 (87–89.5)	89.1 (88–90.3)	0.0006	0.02
	cg12034943	7.4 (6.4–8.4)	7.2 (6.5–8)	0.0007	0.02
	cg17006757	82.8 (80.4–84.2)	83.7 (82–85.3)	0.0008	0.02

Only the sites with significant changes during the first month of treatment are shown. Of note, all significant methylation sites presented in this table are localized in the gene body region of *CRTC1*

β at baseline refers to methylation levels before starting the current psychotropic treatment

β at first month refers to methylation levels after one month of treatment with psychotropic treatment

Controls indicate patients whose weight remained stable during the first month of treatment

Cases indicate patients with important (≥5%) early weight gain during the first month of treatment

*p* values were calculated using paired t-tests. Probes are sorted by significance

^1^*p* values were adjusted using the false discovery rate approach

None: No site with significant change during the first month was observed in control patients

### Association of CRTC1 methylation sites with early weight gain

**Fig. 1:**
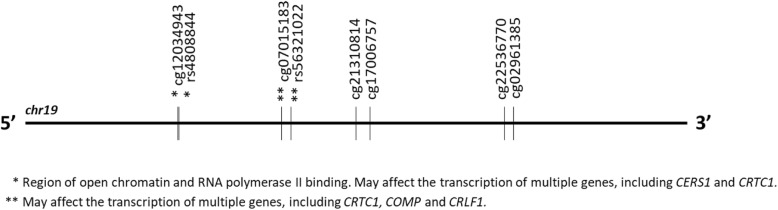
Schematic representation of the significant *CRTC1* methylation sites. The figure is scaled. SNPs associated with methylation sites (i.e., cis-meQTL) are indicated using asterisks. Cis-meQTL: associations between methylation sites and SNPs were extracted from BIOS QTL browser, a public source with available methylation quantitative trait loci (meQTL) data from 3841 Dutch individuals [49]. eQTL: Cis-association data of the influence of SNPs on the expression of nearby genes were extracted from the Genotype-Tissue Expression (GTEx) project, a public source with available expression quantitative trait loci (eQTL) data of 7051 samples from 44 different tissues and for genome-wide genetic variations in the general population (Illumina OMNI 5 M SNP Array) [50]. The functional activity of SNPs was assessed by using the RegulomeDB [51]

The six *CRTC1* methylation probes with significant changes during the first month of treatment (i.e., cg21310814, cg07015183, cg02961385, cg17006757, cg22536770, and cg12034943) (Fig. 1) were tested for association with early weight gain groups.

In particular, multivariable linear mixed models with methylation levels as response adjusting for age, sex, psychotropic drug category, smoking status, treatment duration, and early weight gain group were fitted. In accordance with Additional file 1: Figure S1, Table 3 shows that methylation levels in cg21310814, cg07015183, cg02961385, cg17006757, and cg22536770 increased significantly with treatment duration (*p* ≤ 0.02). In addition, a slight decrease of methylation in cg22536770 was observed with increasing age (*p* = 0.04). In accordance with univariable analysis and as shown in Additional file 1: Figure S2, methylation decrease in cg12034943 during the first month of treatment was significant in cases (*p* = 0.01) but not in control patients (Table 3). The latter was corroborated by an additional regression analysis considering the methylation change as an outcome (calculated as (methylation levels at first month − methylation levels at baseline)/methylation levels at baseline (Additional file 1: Table S2). To summarize, methylation in cg12034943 significantly decreased during the first month of treatment and this decrease was mainly observed in patients with important early weight gain. This suggests that epigenetic change in cg12034943 could be related with psychotropic-induced weight gain.

**Table 3:** Association between *CRTC1* methylation probes and psychotropic induced-early weight gain during the first month of treatment

	cg21310814 (%)		cg07015183		cg02961385 (%)		cg17006757 (%)		cg22536770 (%)		cg12034943 (%)	
	Estimate (SE)	*p* value	Estimate (SE)	*p* value	Estimate (SE)	*p* value	Estimate (SE)	*p* value	Estimate (SE)	*p* value	Estimate (SE)	*p* value
Early weight gain^1^		NS		NS		NS		NS		NS	− 0.3 (0.2)	*0.01*
Age		NS		NS		NS		NS	− 0.008 (0.006)	*0.04*		NS
Sex		NS		NS		NS		NS		NS		NS
Treatment groups^2^		NS		NS		NS		NS		NS		NS
Smoking		NS		NS		NS		NS		NS		NS
Treatment duration^3^	0.05 (0.009)	*0.0001*	0.03 (0.005)	*0.001*	0.02 (0.004)	*< 0.0001*	0.04 (0.008)	*0.02*	0.01 (0.004)	*0.007*		NS

Multivariable mixed models adjusting for age, sex, psychotropic drug category, smoking status, and treatment duration were fitted for 151 observations (from 78 patients). Models on *M* values were conducted to determine the *p* values, whereas models on beta values were conducted to determine the estimates. Validity of multivariable models was verified by plotting residuals against fitted values. Additional multivariable models considering the white blood cell composition were fitted for 68 patients. Neutrophils were significantly associated with cg21310814, cg17006757, and cg12034943 (*p* < 0.0001, *p* = 0.003, and *p* = 0.04). Associations between all *CRTC1* probes and variables indicated in this table remained significant when adjusting for white blood cell composition (except for cg17006757). *p* values in italic are significant

*NS* non-significant

^1^Patients whose weight gain during the first month of treatment was between 0 and 2.5% were considered as controls, whereas patients whose weight gain during the first month of treatment was equal or higher than 5% were considered as cases

^2^Psychotropic drugs were categorized into three groups according to their weight gain propensities: amisulpride and aripiprazole were considered as drugs with a low propensity for weight gain, lithium, mirtazapine, quetiapine, and risperidone were considered as drugs with a moderate propensity for weight gain and clozapine, olanzapine, and valproate were considered as having a high risk for inducing weight gain

^3^Treatment duration was considered in days

### Secondary analyses: association of methylation in cg12034943 with metabolic parameters

In order to further investigate the association between cg12034943 methylation and the pejoration of metabolic parameters during the first month of treatment, additional multivariable models considering metabolic parameters were conducted. In particular, BMI, total cholesterol, low-density lipoprotein cholesterol, high-density lipoprotein cholesterol, triglyceride, and non-high-density lipoprotein cholesterol were tested separately for association with methylation levels in cg12034943. None of these metabolic parameters was associated with cg12034943 methylation during the first month of treatment (Additional file 1: Table S3). In addition, survival analyses showed no influence of this methylation site on overweight incidence (Additional file 1: Table S4). Of note, analyses on metabolic syndrome could not be conducted due to an insufficient number of complete observations (i.e., waist circumference, HDL cholesterol, triglycerides, glycemia, and/or blood pressure).

### Association between rs7258722T>A and cg12034943

**Fig. 2:**
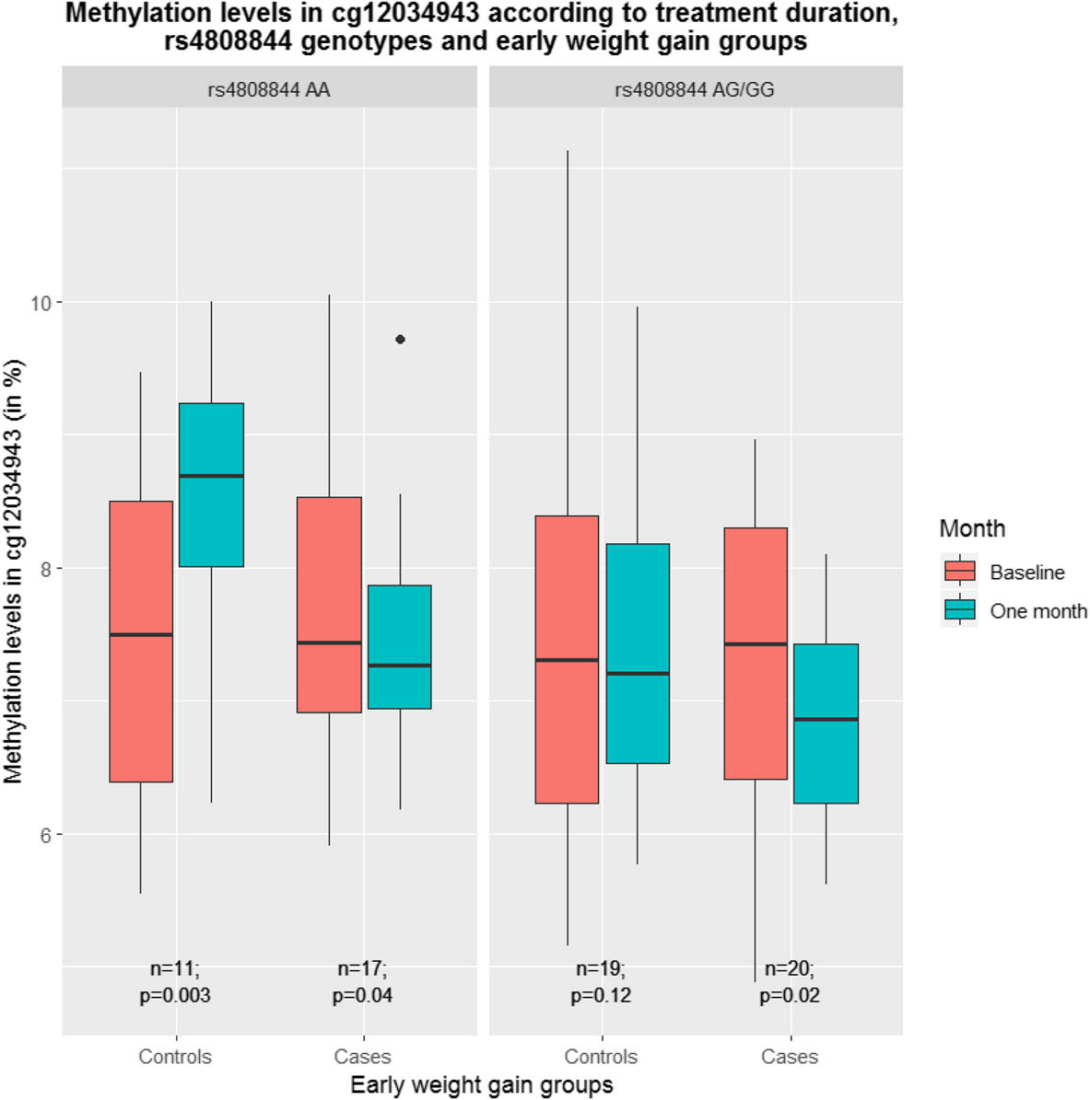
Methylation levels in cg12034943 according to treatment duration, rs4808844 genotypes, and early weight gain groups

According to a recent study considering omics data from blood samples [49], cg12034943 is associated with *CRTC1* rs7258722T>A, with individuals carrying the A-allele of this SNP having lower cg12034943 methylation values as compared to individuals carrying the TT genotype (*p* = 1.8 × 10^−41^). In the present study, similar findings were observed in multivariable models considering a proxy of rs7258722T>A (i.e., rs4808844A>G, *r*^2^ = 0.98) (*p* = 0.03) (Additional file 1: Table S5). Moreover, during the first month of treatment with psychotropic drugs, cg12034943 varied differently depending on the rs4808844A>G genotype. Thus, methylation in cg12034943 was not modified (*p* = 0.9) during the first month of treatment in patients carrying the AA genotype, whereas it decreased significantly (*p* = 0.006) in patients carrying the G allele (Additional file 1: Figure S3). In addition, even when integrating this SNP (which is associated with cg12034943) in multivariable models on methylation outcome, the association between early weight gain and cg12034943 remained significant (Additional file 1: Table S5), suggesting independent and/or synergic influences of early weight gain and rs4808844A>G on cg12034943. When considering observations at the first month of treatment, a trend of interaction (*p* = 0.051) was observed between cg12034943 and rs4808844A>G genotypes on early weight gain groups, as illustrated by the Fig. 2. As a matter of fact, a significant association between early weight gain and cg12034943 was observed in patients carrying the G allele of rs4808844A>G (*p* = 0.03, Additional file 1: Table S5), while no association was identified in patients carrying the rs4808844A>G AA genotype. Early weight gain during treatment with psychotropic drugs therefore probably results from the interaction between genetic (rs4808844A>G) and epigenetic (cg12034943) factors. As rs4808844A>G is associated with cg12034943, this genetic variant was tested for association with early weight gain in a larger and independent sample of patients receiving psychotropic treatments known to induce metabolic disturbances but whose methylation data are not available (*n* = 172). Multivariable logistic regression showed a trend of association between rs4808844A>G and early weight gain, with patients carrying the G allele having less susceptibility of early weight gain as compared to patients carrying the AA genotype (Table 4). In addition, further analyses using generalized additive mixed models on weight in a larger cohort (*n* = 568) without available methylation data showed a trend of association between this SNP and weight, with patients carrying the G allele having lower weight values by 1.55 kg as compared to others (*p* = 0.07; Table 4). However, survival analyses showed no association between rs4808844A>G and overweight and/or obesity incidence in the later cohort (Additional file 1: Table S6).

**Table 4:** Association between rs4808844A>G and weight parameters in an independent psychiatric sample without available methylation data

	Early weight gain^1^			Weight (kg)^2^		
	*n*	Estimate (SE)	*p* value	*n*	Estimate (SE)	*p* value
rs4808844 A>G [G]	131	− 0.66 (0.40)	0.10	568	− 1.55 (1.2)	0.07

^1^A logistic model adjusting for age, sex, psychotropic drug category, smoking status, and treatment duration was fitted in patients with available genetic data for rs4808844 and with available weight at baseline and after the first month of treatment

^2^A GAMM adjusting for age, sex, psychotropic drug category, smoking status, and treatment duration was fitted in patients with available weight and genetic data for rs4808844

## Discussion

The present case-control longitudinal study aimed to determine whether psychotropic drugs could induce methylation modulations in *CRTC1* gene during the first month of treatment and to assess if these methylation changes were associated with an important early weight gain (≥ 5%) during the same period.

Multivariable models showed that methylation decrease in cg12034943 was significantly larger in patients with important weight gain compared to patients whose weight remained stable. This result was corroborated by an independent model considering the relative difference of cg12034943 as an outcome.

During the last few years, a growing number of studies identified associations between certain conditions and methylation quantitative trait loci (meQTLs, i.e., methylation sites associated with genetic variants), which enabled to identify disease-associated transcriptional pathways and to provide possible implications for targeted treatment [52]. Consistent with results from a recent meQTL study performed in the general population [49], cg12034943 was associated with *CRTC1* rs4808844A>G. Furthermore, the modulation of cg12034943 during the first month of treatment was dependent on the rs4808844A>G genotype. In addition, an association between cg12034943 and early weight gain was observed exclusively in patients carrying the rs4808844A>G G allele. Nonetheless, this SNP considered alone was not associated with early weight gain. Therefore, it can be hypothesized that early weight gain during treatment with psychotropic drugs could result from the interaction between genetic and epigenetic factors. Consistent with this assumption, only a trend of association was observed between early weight gain and rs4808844A>G in an independent but much larger psychiatric sample, suggesting that the consideration of epigenetic factors would probably help to increase the observed effect.

To the best of our knowledge, cg12034943 methylation site has been described only once, in a subgroup of the Framingham Heart Study (blood samples from 2567 individuals), where it was associated with symptomatic coronary heart disease [53]. However, no mechanism was proposed and since this study was transversal, no causal inference could be drawn. Most interestingly, in a recent genome-wide association meta-analysis on body fat percentage conducted in more than 100,000 individuals from the general population, *CRTC1* reached genome-wide significance and epigenetic mechanisms were suggested to explain this association [48]. In particular, rs4808844 and rs4808845 were identified as the variants with the greatest amount of regulatory functions, including open chromatin, histone marks that are characteristic of active transcription regulation, and RNA polymerase II binding. Thus, the latter study showed that rs4808844 was significantly associated with RNA polymerase II binding signal strength [48]. In addition, DNaseI hypersensitivity in this genetic region has been shown to correlate negatively with transcription levels of *CRTC1* and of *cytokine receptor like factor 1* (*CRLF1)* in many cell types [54]. Eventually, rs4808844 and its proxies may affect the expression of multiple genes in different tissues, including the *ceramide synthase 1* (*CERS1*) in the adrenal gland [50], a gene which has been involved in the regulation of metabolic features such as obesity-induced insulin resistance [55] and fat metabolism [56]. Taken together, these elements suggest that together with cg12034943, rs4808844 may influence the expression of *CRTC1*, a gene previously described to be associated with metabolic features in the psychiatric population [38, 47].

Although we have not assessed whether the small methylation change of cg12034943 was associated with transcriptional modifications (e.g., of *CRTC1*), small magnitudes of effect (from 2 to 10%, or even smaller [57–60]) resulting from environmental exposures are often observed in environmental epigenetic studies [57] and these small DNA methylation changes may reflect larger changes in chromatin structure and could be associated with broader changes in gene expression [57, 61]. In agreement with that assumption, cg12034943 lies in the intronic region of *CRTC1* and, according to the “regulome” database and to a previous study [48], it is localized in a highly regulated region of open chromatin and in a binding region of a RNA polymerase II. The functional biological significance of methylation change in this genetic region should be assessed by future studies. Of note, although being in a lower range, we can consider that our methylation differences of around 0.7% measured after only a month of treatment in case patients carrying the rs4808844 G allele is consistent with methylation differences observed in previous environmental epigenetic studies that compared life-long methylation differences between exposed versus non-exposed patient groups [57].

Some limitations of the present study should be considered. Even if conducted in longitudinal settings, this study did not allow to determine whether psychotropic drug-induced methylation modulations promoted early weight gain or if psychotropic drug-induced early weight gain promoted the observed methylation change. Such a causal inference could be conducted in future studies using Mendelian randomization, a tool which can be used in this sort of analysis but which usually requires an important sample size (e.g., > 10,000) [62]. In addition, because weight gain induced by psychotropic drugs is mainly mediated by an appetite increase driven by hypothalamic cues, we supposed that the methylation levels observed in blood would reflect methylation changes occurring in the brain. Although brain samples of patients starting a psychotropic medication were not available, a recent study conducted in subjects with medically intractable epilepsy undergoing neurosurgery showed that levels of cg12034943 methylation are comparable in the blood and in the brain [63]. Of note, studies on methylation changes are only feasible using peripheral tissues since postmortem brain samples are available at only one time point, while neurosurgery procedures as in the abovementioned study [63] are expected to occur only once in most if not all cases. It is also noteworthy that, in the present study, continuous BMI was not associated with cg12034943 methylation change. This can probably be explained by our case-control study design, which altered the BMI distribution. Besides, future studies on cg12034943 methylation in patients who lose weight after stopping a psychotropic treatment inducing metabolic disturbances should enable to determine whether change of this methylation site goes in the opposite direction. Moreover, modulations of microRNA levels induced by psychotropic drugs such as antipsychotics [64], mood stabilizers [65–67] and some antidepressants [68] may also be involved in metabolic side effects and should be considered in future studies. Even if a recent study showed that DNA methylation is a better predictor of chronic alterations than RNA expression which seems to fluctuate more in acute conditions [69], research placing emphasis on gene transcription markers would probably help to further understand mechanisms underlying metabolic side effects induced by psychotropic medications.

## Conclusions

In conclusion, the present study identified a methylation change during the first month of psychotropic treatment in the *CRTC1* gene, which was associated with early weight gain during the same period. This epigenetic modulation was dependent on a genetic variant in the same genetic region, which plays a considerable regulatory role. These findings give new insights on psychotropic-induced weight gain and underline the need for future studies considering the combination of genome-wide association studies (GWAS), epigenome-wide association studies (EWAS), and transcription data in order to disentangle the complex mechanisms involved in psychotropic-induced side effects.

## Methods

### Study population

Since 2007, a longitudinal observational study (PsyMetab) has been ongoing at the Department of Psychiatry of the Lausanne University Hospital as described elsewhere [38]. Patients with informed consent starting a psychotropic treatment known to have a potential risk to induce metabolic disturbances (i.e., antipsychotics, mood stabilizers, and some antidepressants, as listed in Additional file 1: Table S7) were included. More details in Additional file 1.

### Case and control patients

A threshold of 5% of weight gain during the first month of treatment was shown to be the best predictor of important weight gain in the longer-term of treatment [70]. Therefore, among PsyMetab patients, those with a weight gain equal or higher than 5% during the first month of treatment were considered as cases, whereas matched (for age, sex, baseline BMI, and medication) PsyMetab patients whose weight remained stable (i.e., weight change between 0 and 2.5%) were considered as controls. Therefore, a total of 39 case and 39 control patients were considered for methylation analyses. Of note, PsyMetab patients with moderate weight loss (e.g., from − 2.5% to 0) were not included in the present study because the observed weight loss could be attributed to a switch from a high risk drug to a drug with a lower propensity for inducing weight gain, which was not of interest for the present study.

### PsyMetab patients without available methylation data

PsyMetab patients whose methylation levels were not available were considered in analyses on the association between a *CRTC1* SNP (rs4808844A>G) and metabolic disturbances induced by psychotropic treatment (e.g., weight gain). Methylation analyses were not conducted in this sample because of a weight loss, an “intermediate” weight gain (between 2.5% and 5%) or because of missing data in at least one important variable to take into account in methylation analyses (age, sex, baseline BMI, medication group, or smoking status).

### DNA methylation array

Two blood samples for each of the 78 case and control patients were collected (i.e., the first was retrieved before starting the psychotropic treatment and the second was drawn 1 month after starting the psychotropic treatment), from which DNA methylation was analyzed using the Illumina Infinium Methylation EPIC BeadChip (Illumina, San Diego, CA, USA). More details are in Additional file 1.

Longitudinal changes in DNA methylation were analyzed for 76 CpG loci in *CRTC1* (as listed in Additional file 1: Table S1), by considering *M* values. The *M* value is calculated as the log2 ratio of the intensities of methylated probe versus unmethylated probe. While a *M* value close to 0 means that the CpG is half-methylated, a positive *M* value means that the CpG has more methylated than unmethylated cytosines and a negative *M* value indicates the opposite ratio [71, 72]. Although the beta-value has a more intuitive biological interpretation, the *M* value is more statistically valid for the differential analysis of methylation levels [72]. Then, as proposed by Du et al. [72], *M* values were used for conducting the differential methylation analyses (and to calculate the *p* values), whereas beta-values were reported in the estimate section of the results.

### Genotyping

A cis-meQTL SNP of interest (i.e., rs4808844A>G) was obtained from the Illumina 200K CardioMetaboChip (Illumina, San Diego, California, USA) [73] at the iGE3 genomics platform of the University of Geneva (http://www.ige3.unige.ch/genomics-platform.php).

### Statistical analysis

Wilcoxon Mann-Whitney rank-sum tests and chi-squared tests were conducted to compare continuous and categorical variables, respectively, across patient groups.

McNemar tests were conducted to evaluate the prevalence differences in certain variables between baseline and the first month of treatment. Paired *t* tests were used to determine the difference of methylation levels between baseline and the first month of treatment. In order to adjust for multiple comparisons (*n* = 76), *p* values were adjusted using the false discovery rate (FDR) approach. Multivariable linear and generalized linear mixed effect models were also used to assess differences among individuals in two groups while adjusting comparisons for potential covariates and cofactors. The fit adequacy was assessed using visual tools. Of note, additional multivariable models adjusting for cell-type composition (i.e., percent neutrophils measured in blood samples) were also conducted. Generalized additive mixed models were used for testing the association between *CRTC1* rs4808844 and weight parameters in an independent psychiatric sample (*n* = 568) of patients starting a psychotropic treatment inducing metabolic disturbances (without available methylation data). These models are powerful and flexible tools useful in capturing highly non-linear trends in time. Functional assessment of methylation sites and SNPs is described in Additional file 1. Statistical significance was defined as a *p* value ≤ 0.05. Statistical analyses were performed using Stata 14 (StataCorp, College Station TX, USA) and R environment for statistical computing version 3.3.1. More details are available in Additional file 1.

## Supplementary information

**Additional file 1.** Supplementary data.

## Data Availability

The datasets used and/or analyzed during the current study are available from the corresponding author on reasonable request.

## Abbreviations

CERS1: Ceramide synthase 1
CRLF1: Cytokine receptor-like factor 1
CRTC1: CREB-regulated transcription coactivator 1
EWAS: Epigenome-wide association studies
GWAS: Genome-wide association studies
meQTL: Methylation quantitative trait loci
SNPs: Single nucleotide polymorphisms
