# Supplementary material for: Psychotropic drug-induced genetic-epigenetic modulation of CRTC1 gene is associated with early weight gain in a prospective study of psychiatric patients
Source: Clin Epigenetics. 2019 Dec 26;11:198. doi: 10.1186/s13148-019-0792-0 (PMC6933694; doi:10.1186/s13148-019-0792-0)
Supplement: Supplementary file 1 — Additional file 1. Supplementary data. [file 13148_2019_792_MOESM1_ESM.docx]

**SUPPLEMENTARY DATA**

**Study population**

Data were obtained in the hospital or in outpatients centers during a medical examination based on a departmental guideline for metabolic follow-up performed on a routine basis (1). Monitoring for physical health risk factors include prospective assessments of body mass index (BMI), waist circumference, fasting glucose, lipid profile, blood pressure, and tobacco smoking during treatment (2). When a treatment was stopped for more than 2 weeks or if a drug was replaced by another drug on the list, the follow-up was restarted from baseline. In case of the introduction of a second studied drug, the follow-up was restarted and the last introduced drug was considered as the main treatment. This study was approved by the Ethics Committee of the Lausanne University Hospital.

**DNA methylation array**

DNA was extracted using the Flexigene DNA Kit or QIAamp DNA Blood Mini QIAcubee Kit (Qiagen AG, Switzerland) as described by the manufacturer’s instructions. DNA was bisulfite-converted using the EZ-96 DNA Methylation Kit (Zymo Research Corporation, Irvine, USA) as recommended by the manufacturer. DNA methylation was assessed using the Illumina Infinium Methylation EPIC BeadChip (Illumina, San Diego, CA, USA), which interrogates over than 850,000 methylation sites per sample at single-site nucleotide resolution. Analyses were performed at the iGE3 genomics platform of the University of Geneva (<http://www.ige3.unige.ch/genomics-platform.php>). Baseline and corresponding follow-up samples were analyzed on the same array and, in order to avoid batch effect and other potential technical sources of variation, samples from case and control patients were randomly assigned to the different batches.

**Quality control**

The different types of controls (i.e. for staining, extension, hybridization, target removal, G/T mismatch, bisulfite conversion, specificity, negative and non-polymorphic) were visualized in GenomeStudio. Additional probe and sample quality controls were conducted in R environment for statistical computing version 3.3.1 by the Swiss Institute of Bioinformatics (SIB) (https://www.sib.swiss). 8131 probes for which at least 5% of samples had a detection P-value greater than 0.01 were removed. Then, the remaining 857’728 CpG sites passed the quality control steps and were eligible for further analysis. In the last step, data were normalized by Subset-Quantile Within Array Normalization (SWAN), using the minfi package (3).

**Functional assessment of methylation sites and SNPs**

*Cis-meQTL analysis*

Cis-associations between SNPs and methylation sites were extracted from BIOS QTL browser, a public source (<https://genenetwork.nl/biosqtlbrowser/>) with available methylation quantitative trait loci (meQTL) data from 3841 Dutch individuals(4).

*eQTL analysis*

Cis-association data of the influence of SNPs on the expression of nearby genes were extracted from the Genotype-Tissue Expression (GTEx) project, a public source (<http://www.gtexportal.org/home/>) with available expression Quantative Trait Loci (eQTL) data of 7051 samples from 44 different tissues and for genome-wide genetic variations in the general population (Illumina OMNI 5M SNP Array) (5).

*RegulomeDB annotation*

The Regulome database (http://www.regulomedb.org/) was used to assess the functional activity of significant SNPs (6). This database defines SNPs with known and/or predicted regulatory elements located in intergenic regions of the human genome. Known and predicted regulatory elements include regions of DNAase hypersensitivity, binding sites of transcription factors and promoter regions biochemically characterized to the regulation of transcription. Sources for these data include public datasets from GEO, the ENCODE project and the published literature.

**Statistical analyses**

Only the *CRTC1* methylation probes with significant change during the first month of treatment were tested for association with early weight gain in multivariate analyses. In particular, linear mixed models on cg21310814, cg07015183, cg02961385, cg17006757, cg22536770 and cg12034943 adjusting for age, sex, psychotropic drug category, smoking status and treatment duration were fitted for 151 observations (from 78 patients), using the nlme package of R. Of note, smoking status was not available for 5 observations. As previously mentioned, models on M-values were conducted to determine the p-values, whereas models on beta values were conducted to determine the estimates. Kaplan-Meier with log-rank tests and Cox regression tests adjusting for age, sex, psychotropic drug category and smoking status were used to compare the incidence of overweight and obesity development according to early methylation changes and/or to rs4808844 genotype, using the survival package of R. The association between early weight gain and rs4808844 genotype was assessed using a logistic regression adjusting for age, sex, psychotropic drug category, smoking status and treatment duration, in patients with available genetic data for rs4808844 and with available weight at baseline and after the first month of treatment.

1. Choong E, Solida A, Lechaire C, Conus P, Eap CB. Follow-up of the metabolic syndrome induced by atypical antipsychotics: recommendations and pharmacogenetics perspectives. Revue medicale suisse. 2008;4(171):1994-9.

2. Cooper SJ, Reynolds GP, Barnes T, England E, Haddad PM, Heald A, et al. BAP guidelines on the management of weight gain, metabolic disturbances and cardiovascular risk associated with psychosis and antipsychotic drug treatment. Journal of psychopharmacology (Oxford, England). 2016;30(8):717-48.

3. Maksimovic J, Gordon L, Oshlack A. SWAN: Subset-quantile within array normalization for illumina infinium HumanMethylation450 BeadChips. Genome biology. 2012;13(6):R44.

4. Bonder MJ, Luijk R, Zhernakova DV, Moed M, Deelen P, Vermaat M, et al. Disease variants alter transcription factor levels and methylation of their binding sites. Nature genetics. 2017;49(1):131-8.

5. GTEx Consortium. The Genotype-Tissue Expression (GTEx) project. Nature genetics. 2013;45(6):580-5.

6. Boyle AP, Hong EL, Hariharan M, Cheng Y, Schaub MA, Kasowski M, et al. Annotation of functional variation in personal genomes using RegulomeDB. Genome research. 2012;22(9):1790-7.

**Table S1. List of *CRTC1* methylation sites analyzed.**

Probes are sorted by genomic positions. *CRTC1* gene is 98'719 base pairs long, localizing from chr19:18683615 to ch19:18782333 (GRCh38).

**Table S2. Association between cg12034943 change and psychotropic induced-early weight gain during the first month of treatment.**

Multivariate models adjusting for age, sex, psychotropic drug category, smoking status and treatment duration were fitted for 78 patients. Model on M-values were conducted to determine p-values, whereas model on beta values were conducted to determine estimates.

^1^ Δ cg12034943 was calculated as follow: (cg12034943 levels at first month - cg12034943 levels at baseline) / cg12034943 levels at baseline.

^2^ Patients whose weight gain during the first month of treatment was between 0 and 2.5% were considered as controls, whereas patients whose weight gain during the first month of treatment was equal or higher than 5% were considered as cases.

^3^ Psychotropic drugs were categorized into three groups according to their weight gain propensities: amisulpride and aripiprazole were considered as drugs with a low propensity for weight gain, lithium, mirtazapine, quetiapine and risperidone were considered as drugs with a moderate propensity for weight gain and clozapine, olanzapine and valproate were considered as having a high risk for inducing weight gain.

^4^ Treatment duration was considered in days.

Abbreviation: NS: non significant.

P-values in bold are significant.

Validity of this multivariate model was verified by plotting residuals against fitted values.

An additional multivariate model considering the white blood cell composition were fitted for 61 patients. Reported associations (i.e. between early weight gain and cg12034943 as well as between sex and cg12034943) remained significant when adjusting for white blood cell composition.

**Table S3. Association between cg12034943 and metabolic variables during the first month of treatment.**

Multivariable mixed models adjusting for age, sex, psychotropic drug category, smoking status and treatment duration were fitted. Models on M-values were conducted to determine the p-values, whereas models on beta values were conducted to determine the estimates.

^1^ Analyses on total cholesterol, HDL-cholesterol, LDL-cholesterol, non-HDL cholesterol and triglyceride were conducted only in patients with no prescription of any lipid lowering drug prescribed in the present metabolic follow-up (i.e. atorvastatin, ezetimibe, fenofibrate, fluvastatin, pravastatin, rosuvastatin or simvastatin).

^2^ Psychotropic drugs were categorized into three groups according to their weight gain propensities: amisulpride and aripiprazole were considered as drugs with a low propensity for weight gain, lithium, mirtazapine, quetiapine and risperidone were considered as drugs with a moderate propensity for weight gain and clozapine, olanzapine and valproate were considered as having a high risk for inducing weight gain.

^3^ Treatment duration was considered in days.

Abbreviation: NS: non significant.

Validity of multivariate models was verified. Of note, analyses on metabolic syndrome could not been conducted due to an insufficient number of complete observations (i.e waist circumference, HDL cholesterol, triglycerides, glycemia and/or blood pressure).

**Table S4. Risk factors for new onset overweight in patients receiving psychotropic treatment inducing metabolic disturbances**

Results were obtained by fitting a Cox regression controlling for age, gender, psychotropic drug category, smoking status and the change of methylation in cg12034943 during the first month of treatment in 47 patients.

Among the 47 patients, 5 developed overweight during the first month of psychotropic treatment.

^1^ Psychotropic drugs were categorized into three groups according to their weight gain propensities: amisulpride and aripiprazole were considered as drugs with a low propensity for weight gain, lithium, mirtazapine, quetiapine and risperidone were considered as drugs with a moderate propensity for weight gain and clozapine, olanzapine and valproate were considered as having a high risk for inducing weight gain.

^2^ Δcg12034943 was calculated as follow: (cg12034943 levels at first month - cg12034943 levels at baseline) / cg12034943 levels at baseline.

P-value in bold is significant.

**Table S5. Association between cg12034943 and rs4808844 in the overall sample and in rs4808844 stratified groups.**

Multivariate mixed models adjusting for age, sex, psychotropic drug category, smoking status, treatment duration, rs4808844 genotype and early weight gain groups were fitted for 67 patients. Models on M-values were conducted to determine the p-values, whereas models on beta values were conducted to determine the estimates.

^1^ Patients whose weight gain during the first month of treatment was between 0 and 2.5% were considered as controls, whereas patients whose weight gain during the first month of treatment was equal or higher than 5% were considered as cases.

^2^ Psychotropic drugs were categorized into three groups according to their weight gain propensities: amisulpride and aripiprazole were considered as drugs with a low propensity for weight gain, lithium, mirtazapine, quetiapine and risperidone were considered as drugs with a moderate propensity for weight gain and clozapine, olanzapine and valproate were considered as having a high risk for inducing weight gain.

^3^ Treatment duration was considered in days.

^4^ Patients carrying the G allele were compared to patients carrying the AA genotype.

Abbreviation: NS: non significant.

P-values in bold are significant.

Validity of multivariate models was verified by plotting residuals against fitted values.

**Table S6. Risk factors for new onset overweight in an independent sample of patients receiving psychotropic treatment inducing metabolic disturbances**

Results were obtained by fitting a Cox regression controlling for age, gender, psychotropic drug category, smoking status and rs4808844 in 349 patients for new onset overweight and in 480 patients for new onset obesity.

During the psychotropic treatment, 77 out of the 349 patients developed overweight and 48 out of the 480 patients developed obesity.

^1^ Psychotropic drugs were categorized into three groups according to their weight gain propensities: amisulpride, aripiprazole, haloperidol and zuclopenthixol were considered as drugs with a low propensity for weight gain, lithium, mirtazapine, quetiapine and risperidone were considered as drugs with a moderate propensity for weight gain and clozapine, olanzapine and valproate were considered as having a high risk for inducing weight gain.

^2^ Patients carrying the G allele were compared to patients carrying the AA genotype.

**Table S7. Drugs included in the metabolic follow-up recommendation**

| **ANTIPSYCHOTICS** | | **ANTIDEPRESSANTS** | | **MOOD STABILIZERS** |
| --- | --- | --- | --- | --- |
|  |  |  |  |  |
| **Atypical  (second-generation)** | **Typical  (first-generation)** | **Tricyclic** | **Other** |  |
|  |  |  |  |  |
| Amisulpride | Chlorprothixene | Amitriptyline | Mirtazapine | Carbamazepine |
| Aripiprazole | Flupentixol | Clomipramine |  | Lithium |
| Asenapine | Haloperidol | Doxepine |  | Valproate |
| Clozapine | Levomepromazine | Imipramine |  |  |
| Lurasidone | Pipamperone | Nortriptyline |  |  |
| Olanzapine | Promazine | Opipramol |  |  |
| Paliperidone | Sulpiride | Trimipramine |  |  |
| Quetiapine | Tiapride |  |  |  |
| Risperidone | Zuclopenthixol |  |  |  |
| Sertindole |  |  |  |  |
|  |  |  |  |  |

According to international recommendations, a metabolic follow-up is ongoing since 2007 at the Department of Psychiatry in the Lausanne University Hospital (1), where inpatients and outpatients are prospectively monitored when starting a pharmacological treatment known to have a potential risk to induce metabolic disturbances (i.e. drugs listed above).

1. Choong E, Solida A, Lechaire C, Conus P, Eap CB. Follow-up of the metabolic syndrome induced by atypical antipsychotics: recommendations and pharmacogenetics perspectives. Revue medicale suisse. 2008;4(171):1994-9.

2. Cooper SJ, Reynolds GP, Barnes T, England E, Haddad PM, Heald A, et al. BAP guidelines on the management of weight gain, metabolic disturbances and cardiovascular risk associated with psychosis and antipsychotic drug treatment. Journal of psychopharmacology (Oxford, England). 2016;30(8):717-48.

3. Maksimovic J, Gordon L, Oshlack A. SWAN: Subset-quantile within array normalization for illumina infinium HumanMethylation450 BeadChips. Genome biology. 2012;13(6):R44.

4. Bonder MJ, Luijk R, Zhernakova DV, Moed M, Deelen P, Vermaat M, et al. Disease variants alter transcription factor levels and methylation of their binding sites. Nature genetics. 2017;49(1):131-8.

5. GTEx Consortium. The Genotype-Tissue Expression (GTEx) project. Nature genetics. 2013;45(6):580-5.

6. Boyle AP, Hong EL, Hariharan M, Cheng Y, Schaub MA, Kasowski M, et al. Annotation of functional variation in personal genomes using RegulomeDB. Genome research. 2012;22(9):1790-7.


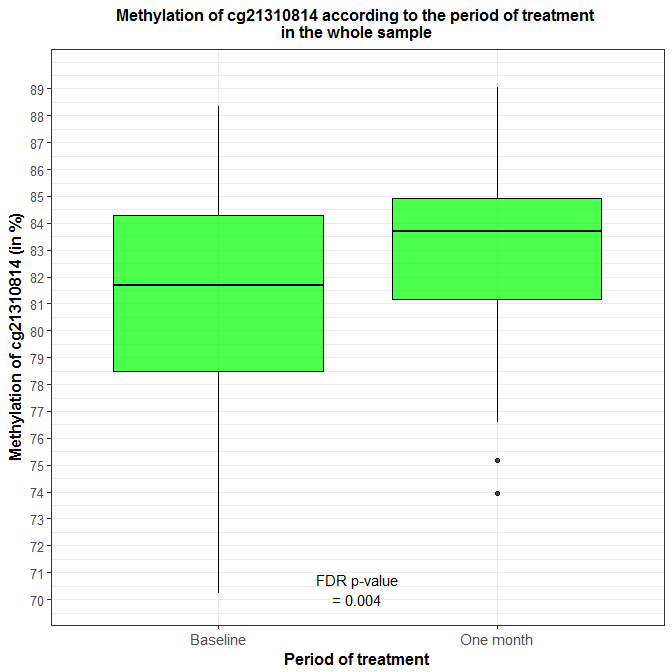


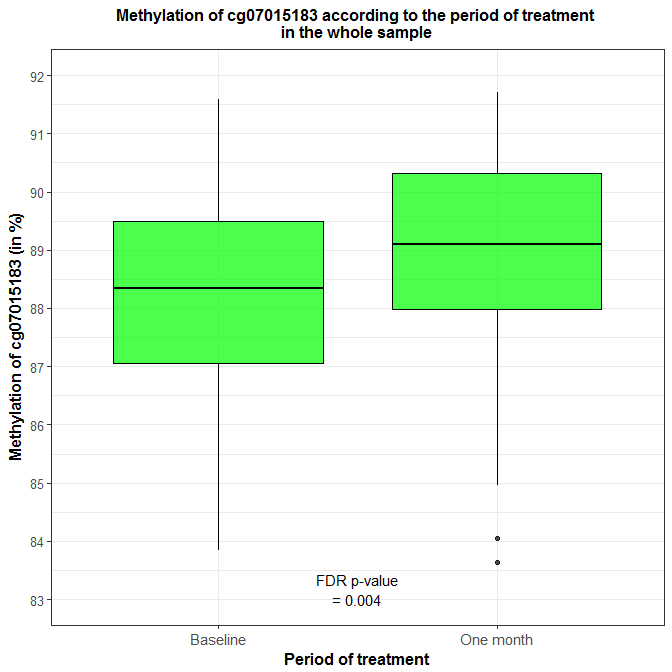


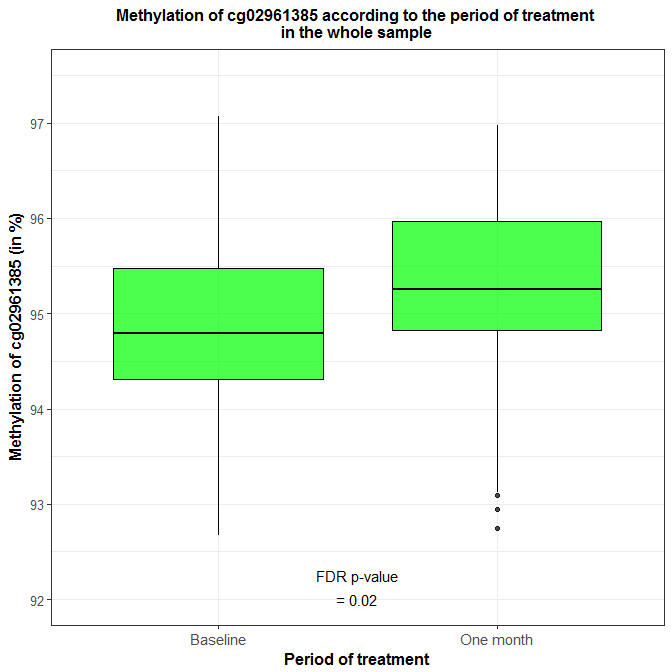


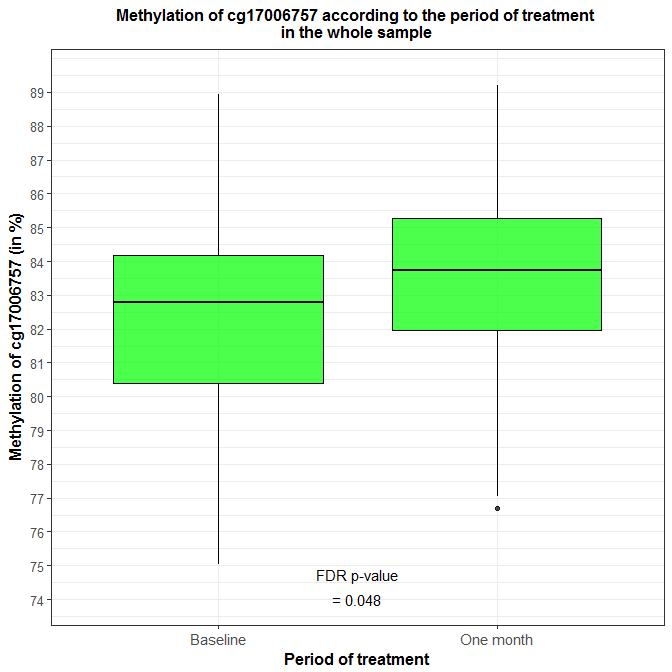


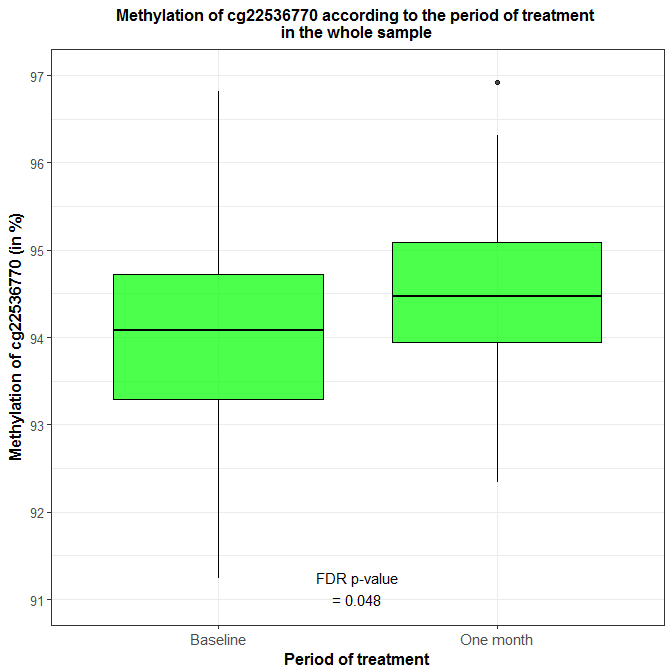


**Figure S1 A.**

*CRTC1* methylation levels according to the period of psychotropic treatment in the whole sample (n=78).


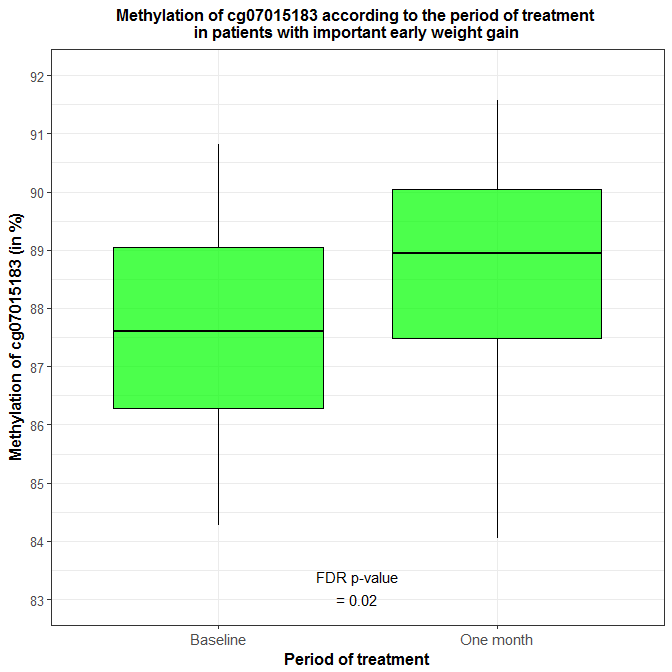


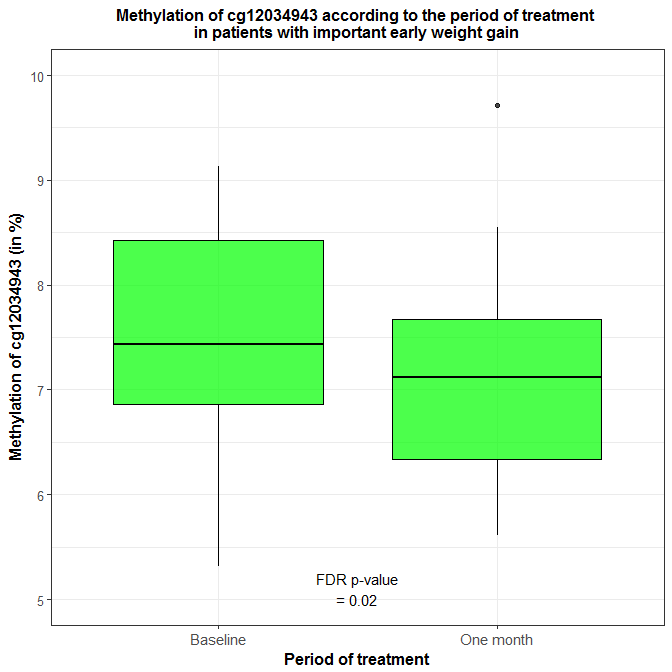


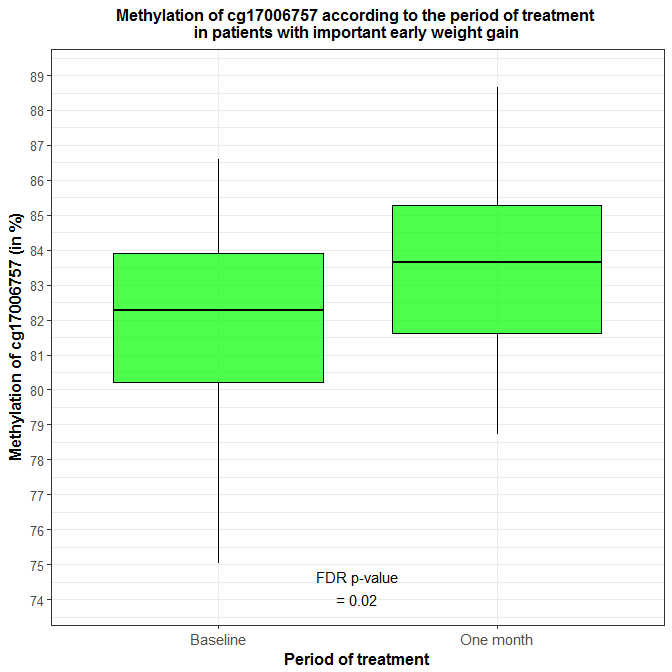


**Figure S1 B.**

*CRTC1* methylation levels according to the period of psychotropic treatment in patients with important early weight gain (n=39).


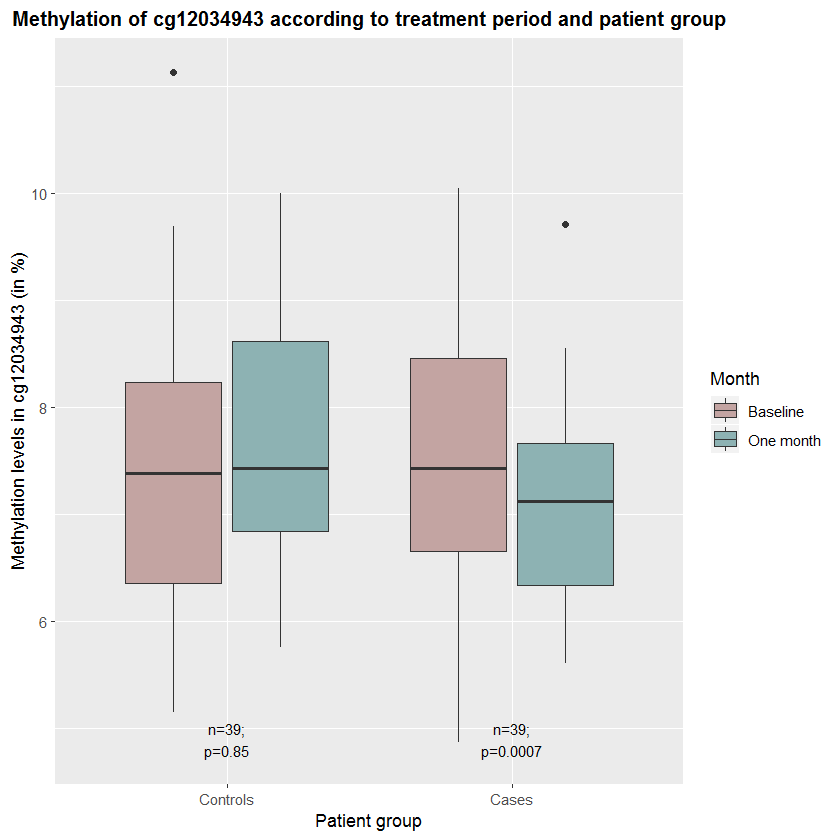


**Figure S2. Methylation levels of cg12034943 according to treatment duration and patient group.**


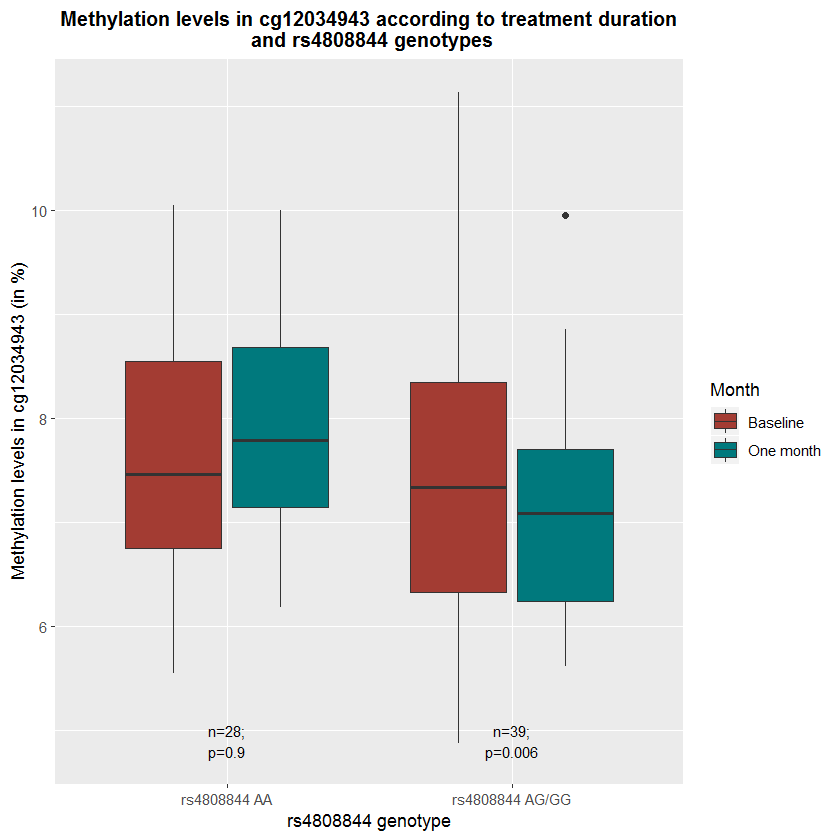


**Figure S3. Methylation levels in cg12034943 according to treatment duration and rs4808844 genotypes.**
